# Why are heart operations postponed?

**DOI:** 10.1186/1749-8090-6-106

**Published:** 2011-09-05

**Authors:** Georgios I Tagarakis, Dimos Karangelis, Christos Voucharas, Marios E Daskalopoulos, Theocharis Koufakis, Maria Mouzaki, Stefania Lampoura, Dimitrios Papadopoulos, Ilias Sataitidis, Nikolaos B Tsilimingas

**Affiliations:** 1 Department of Cardiovascular and Thoracic Surgery, University of Thessaly, Larissa, Greece

## Abstract

**Aim:**

To investigate the reasons that lead to postponement of cardiac operations, in order to elucidate the problem and help patients through modes of prevention.

**Methods-Design:**

We retrospectively included in the study all patients submitted to elective adult heart surgery in our department during the 4-year period 2007-2010 and noted all cases of postponement after official inclusion in the operating schedule.

**Results:**

94 out of a total of 575 patients (16.34%) scheduled for elective cardiac operation had their procedure postponed. The reasons were mainly organisatory (in 49 cases, 52.12%), which in order of significance were: unavailability in operating rooms, shortage in matching erythrocyte units and shortage in anaesthetic/nursing staff. The rest of the cases (45, 47.88%) were postponed due to medical reasons, which in order of significance were: febrile situations, including infections of the respiratory, gastrointestinal and urinary system, problems with the regulation of antiplatelet and antithrombotic drugs, neurological manifestations such as stroke and transient ischaemic attacks, exacerbation of asthma/chronic obstructive pulmonary disease, arrhythmias, renal problems and allergic reactions to drugs. Patients with advanced age and increased Euroscore values were most possible to have their heart operation postponed.

**Conclusions:**

Heart operations are postponed due to organisatory as well as medical reasons, the latter mainly affecting older, morbid patients who therefore require advanced preoperative care.

## Introduction

Every physician employed in the practice of heart surgery is aware of the fact that the psychological condition of a heart patient scheduled for cardiac surgery is a very fragile one. Such patients have already been burdened with multiple examinations and hospital admissions and the vast majority of them suffers from a variety of other, non cardiac problems. In this setting, heart operation appears as a last great obstacle that can be overcome only if the remaining psychological resources of the patient are recruited. One can easily imagine the magnitude of stress such a patient experiences when his heart operation is postponed [1,2].

In this study we are presenting the experience of our department on the matter during the last 4-year period by analyzing the reasons that led to the postponement of cardiac operations. Although organisatory reasons have also been taken into account, we have mainly focused on the medical conditions that are responsible for this postponement.

## Methods and design

We retrospectively included in this epidemiologic analysis all patients scheduled and prepared for elective heart surgery in our department in the 4-year period 2007-2010, whose operation was postponed for any reason. We excluded patients with urgent operation who are entering the operating room even if not all required parameters of the preoperative preparation have been fulfilled and whose operation is practically never postponed. The aforementioned criteria led to the inclusion in the study of 575 out of a total of 728 heart patients (78.9%) operated in our department during the same period.

As far as the characteristics of our department are concerned: it is a cardiovascular and thoracic surgery department of a university tertiary care hospital, operating with European Union standards, covering with every-day 24-hour duty an area of responsibility of 1.3 million inhabitants. This explains some of the organisatory problems that arise in everyday practice.

## Results

In the examined period 94 patients (94/575 = 16.34%) had their elective heart operation postponed. In 49 of the cases the main reason was organisatory (Table 1), more specifically: i) 25 cases (51%) unavailability of operating rooms which were given to more urgent cases, ii) 14 cases (28.57%) shortage of matching erythrocyte units which were given to more urgent cases, although initially provided for the heart-operated patient iii) 10 cases (20.4%) shortage in anaesthetic or nursing staff owed to sudden illness and absence from work. In the rest 45 cases (7.82% of the total of patients) the reason for the postponement were medical conditions that would jeopardize the safe course of the operation if left untreated (Table 1). These were noted in descending order of frequency as follows: i) febrile conditions in 17 cases (37.77%) of which 11 with respiratory infection, 3 with gastrointestinal infection and 3 with urinary tract infection ii) 13 cases (28.8%) with misinterpretation/incompliance of the patient with the medical order to discontinue antiplatelet/antithrombotic agents iii) 4 patients (8.88%) with neurological manifestations, including one patient with stroke and three with TIA iv) 4 patients (8.88%) with exacerbation of asthma/chronic obstructive pulmonary disease iv) three patients (6.67%) with cardiac problems (arrhythmias in form of atrial fibrillation) that required stabilization prior to surgery, v) two patients (4.44%) with increased blood urea/creatinine values during the last preoperative check, who were therefore scheduled for nephrological consultation vi) two patients (4.44%) with allergic reaction to newly administered drugs.

**Table 1:** Analysis of organisatory problems and medical conditions responsible for postponement of elective heart operations

Organisatory, n = 49 (51.57%)	Medical n = 45 (47.36%)
	
	17 patients with febrile conditions (37.77%) (11 respiratory infection, 3 gastrointestinal infection, 3 urinary tract infection)
Unavailability in operating rooms 25 (51%)	13 patients (28.8%) with misinterpretation/incompliance of the patient with the medical order to discontinue antiplatelet/antithrombotic agents

Shortage in matching erythrocyte units 14 (28.57%)	4 patients (8.88%) with neurological manifestations, including one patient with stroke and three with TIA

Shortage in anaesthetic/nursing staff 10 (20.4%)	4 patients (8.88%) with exacerbation of asthma/chronic obstructive pulmonary disease
	
	3 patients (6.67%) with cardiac manifestations (arrhythmias in form of atrial fibrillation) that required stabilization prior to surgery
	
	two patients (4.44%) with increased blood urea/creatinine values during the last preoperative check, who were therefore scheduled for nephrological consultation
	
	two patients (4.44%) with allergic reaction to recently administered drugs

The table depicts in details all reasons, both organisatory as well as medical, that lead to the postponement of heart operations.

Seven of the patients with postponement due to organisatory reasons had their operation postponed for the same reason for a second time. All of the patients who were postponed for medical conditions were operated with a delay ranging from 3 days for simpler conditions such as allergic reactions to 10 days for more grave conditions, such as persisting respiratory infections.

To answer the question 'what was the profile of the patients whose operation was postponed for medical reasons", we noted that advanced age and elevated values in Euroscore [3,4] seemed to correlate with augmented possibilities towards postponement, while gender or type of scheduled procedure appeared to be insignificant (mean age of postponed patients 72.3 vs 67.2 of the rest of the patients p < 0.01, mean Euroscore (patient- and cardiac related parameters) value of postponed patients 14.6 (SD ± 1.4) vs 10.1 (SD ± 0.9) of the rest of the patients, p < 0.01, male gender 66 out of 95 (70.21%) vs 342 out of 481 (71.1%), CABG 68 (72.3%) vs 356 (74%), aortic valve surgery 10 (10.63%) vs 47 (9.77%), mitral valve surgery 7 (7.44%) vs 30 (6.23%), combined procedures 6 (6.38%) vs 34 (7.06%) aortic surgery 3 (3.19%) vs 14 (2.91%) (Table 2).

**Table 2:** Comparative analysis of demographic and medical parameters between patients with postponed and those without postponed cardiac procedure

	Postponed Patients N = 94	Non Postponed Patients n = 481	Statistical Significance
Age	72.3	67.2	p < 0.01

Gender	66 (70.21%)	342 (71.1%)	non significant

Mean Euroscore (patient-and cardiac related parameters)	14.6 (SD ± 1.4)	10.1 (SD ± 0.9)	p < 0.01

Scheduled for CABG	68 (72.3%)	356 (74%)	non significant

Scheduled for Aortic Valve Surgery	10 (10.63%)	vs 47 (9.77%)	non significant

Scheduled for Mitral Valve Surgery	7 (7.44%)	30 (6.23%),	non significant

Scheduled for Combined Surgery	6 (6.38%)	34 (7.06%)	non significant

Scheduled for Aortic Surgery	3 (3.19%)	14 (2.91%)	non significant

The table shows comparative analysis of demographic and medical parameters between patients with and those without postponed operations. The x2 criterion was applied for categorical parameters, the t-student (unpaired) for continuous ones. The Kolmogorov-Smirnoff test was used for the evaluation of normal distribution of samples. The level of statistical significance was set at a level for p < 0.05.

## Discussion

This study deals with the important issue of postponement of heart operations, a situation that causes both psychological burden for the patients as well as augmented hospitalization costs for any health system. To the best of our knowledge this is one of the few (three) of the kind in medical literature and the one based on the broadest sample. The study was conducted in a tertiary care university department with 24-hour/days emergency duty responsibility, a fact that can explain some of the organisatory problems encountered. The study concluded to the following results.

First of all, the majority of cardiac operations are postponed due to administrative/organisatory reasons. This makes, among other measures, imperative the need for better management and better use of resources for the sake of the patients, but also for reasons of financing and economy. The study referred to data from a period where both Greece as well as European Union faced serious financial recession, a fact that can cause augmented organisatory problems through lack cuts in expenses in infrastructure, material and employment of specialized personnel.

Attending physicians and nursing staff should be aware of the medical reasons that usually lead to the postponement of cardiac operations in an effort to avoid them: preooperative infections, especially of the respiratory system, wrong and prolonged used of antiplatelet/antithrombotic agents, neurological conditions, exacerbation of COPD and asthma, arrhythmias and allergic reactions to drugs are the most important.

These conditions most easily tend to affect old multimorbid patients, whose preoperative care should therefore be of the best possible quality. Hopefully and, against the difficulties of the global economic crisis, the organization of health units will allow in the future a better standard of care for cardiac patients scheduled for heart surgery, so as to avoid psychologically painful and economically burdening cases of operation postponement.

## Conflicts of interest statement

The authors declare that they have no competing interests.

## Authors' contributions

GT was the main author. DK co-authored the paper. CV performed literature research. MD performed linguistic control. TC performed the statistical analysis. MM performed literature research. SL performed a final check of the manuscript. DP was member of the anaesthetic team and checked the paper. IS was a member of the surgical team and checked the paper. NT was the head of the department and made the final check of the paper. All authors have read and approved the final manuscript
